# Minimum Reporting Standards for in vivo Magnetic Resonance Spectroscopy (MRSinMRS): Experts' consensus recommendations

**DOI:** 10.1002/nbm.4484

**Published:** 2021-02-09

**Authors:** Alexander Lin, Ovidiu Andronesi, Wolfgang Bogner, In‐Young Choi, Eduardo Coello, Cristina Cudalbu, Christoph Juchem, Graham J. Kemp, Roland Kreis, Martin Krššák, Phil Lee, Andrew A. Maudsley, Martin Meyerspeer, Vladamir Mlynarik, Jamie Near, Gülin Öz, Aimie L. Peek, Nicolaas A. Puts, Eva‐Maria Ratai, Ivan Tkáč, Paul G. Mullins, Experts' Working Group on Reporting Standards for MR Spectroscopy

**Affiliations:** ^1^ Center for Clinical Spectroscopy, Department of Radiology, Brigham and Women's Hospital Harvard Medical School Boston Massachusetts USA; ^2^ Department of Radiology Massachusetts General Hospital Boston Massachusetts USA; ^3^ High Field MR Center, Department of Biomedical Imaging and Image‐guided Therapy Medical University of Vienna Vienna Austria; ^4^ Department of Neurology, Hoglund Biomedical Imaging Center University of Kansas Medical Center Kansas City Kansas USA; ^5^ Center for Biomedical Imaging (CIBM), Ecole Polytechnique Fédérale de Lausanne Lausanne Switzerland; ^6^ Departments of Biomedical Engineering and Radiology Columbia University New York New York USA; ^7^ Department of Musculoskeletal and Ageing Science and Liverpool Magnetic Resonance Imaging Centre (LiMRIC) University of Liverpool Liverpool UK; ^8^ Departments of Radiology and Biomedical Research University of Bern Bern Switzerland; ^9^ Department of Medicine III and Department of Biomedical Imaging and Image guided Therapy Medical University of Vienna Vienna Austria; ^10^ Department of Radiology, Hoglund Biomedical Imaging Center University of Kansas Medical Center Kansas City Kansas USA; ^11^ Department of Radiology University of Miami Coral Gables Florida USA; ^12^ High Field MR Center, Center for Medical Physics and Biomedical Engineering Medical University of Vienna Vienna Austria; ^13^ Magnetic Resonance Centre of Excellence. Medical University of Vienna Vienna Austria; ^14^ Brain Imaging Centre, Douglas Research Centre, Department of Psychiatry McGill University Montreal Quebec Canada; ^15^ Center for Magnetic Resonance Research, Department of Radiology University of Minnesota Minneapolis Minnesota USA; ^16^ Faculty of Health Sciences University of Sydney Sydney Australia; ^17^ Department of Forensic and Neurodevelopmental Sciences Sackler Institute for Translational Neurodevelopment, Institute of Psychiatry, Psychology, and Neuroscience, King's College London London UK; ^18^ A.A. Martinos Center for Biomedical Imaging, Neuroradiology Division, Department of Radiology Massachusetts General Hospital Boston Massachusetts USA; ^19^ Bangor Imaging Unit, School of Psychology Bangor University Bangor Gwynedd UK

**Keywords:** MR spectroscopy (MRS) and spectroscopic imaging (MRSI) methods, reporting guidelines

## Abstract

The translation of MRS to clinical practice has been impeded by the lack of technical standardization. There are multiple methods of acquisition, post‐processing, and analysis whose details greatly impact the interpretation of the results. These details are often not fully reported, making it difficult to assess MRS studies on a standardized basis. This hampers the reviewing of manuscripts, limits the reproducibility of study results, and complicates meta‐analysis of the literature. In this paper a consensus group of MRS experts provides minimum guidelines for the reporting of MRS methods and results, including the standardized description of MRS hardware, data acquisition, analysis, and quality assessment. This consensus statement describes each of these requirements in detail and includes a checklist to assist authors and journal reviewers and to provide a practical way for journal editors to ensure that MRS studies are reported in full.

## Graphical abstract

This paper sets out the expert consensus on minimum reporting guidelines for MRS studies. It includes a checklist as a practical way for authors and journal editors to ensure that MRS studies are reported appropriately. A common set of reporting standards will greatly increase transparency, rigor, and replicability of MRS studies, and should further the successful integration of MRS into widespread clinical practice.

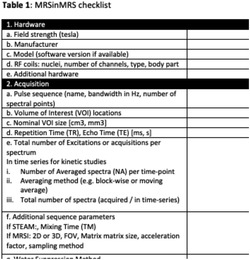

**Abbreviations**
^1^H MRS: proton MRS
2D: two dimensional
3D: three dimensional
CRLB: Cramér‐Rao lower bound
FOV: field of view
FWHM: full‐width at half‐maximum
MRSI: magnetic resonance spectroscopic imaging
NA: number of acquisitions per spectrum
NAA: *N*‐acetylaspartate
ppm: parts per million
PRESS: point resolved spectroscopy
SD: standard deviation
SNR: signal‐to‐noise ratio
STEAM: stimulated echo acquisition mode
tCho: total choline
tCr: total creatine
*T*_E_
: echo time
*T*_E1_
: first sub‐echo time in PRESS sequence
*T*_E2_
: second sub‐echo time in PRESS sequence
*T*_M_
: mixing time
*T*_R_
: repetition time
VOI: volume of interest

## 1 INTRODUCTION

Despite over 30 years of development and thousands of papers describing the use of in vivo MRS for non‐invasive research in health and disease, including diagnosis and treatment monitoring across a broad range of human conditions, MRS has yet to reach full clinical acceptance.^1^ While there remain several important technical issues,^2^ one of the major problems is the lack of standards for reporting MRS studies. The importance can be described on several levels. First, there is increasing concern in the general scientific community over the lack of rigor and reproducibility of scientific studies.^3^ Details of MRS methodologies need to be fully reported for readers to critically evaluate the quality of the published results and to reproduce the experiments. Second, recent meta‐analyses and evidence‐based reviews of MRS^4^ have noted the lack of detail in peer‐reviewed publications, which makes it difficult to compare study results. Third, the lack of reporting guidelines for MRS means that new researchers in the field find limited guidance on practice. As a result, MRS studies are sometimes conducted using inappropriate or incorrect methods that may lead to erroneous and/or inconsistent conclusions. Finally, MRS is a versatile method that finds application across fields where there may be insufficient peer expertise to provide critical technical evaluation of methods and analysis. A core set of standards for the rigorous reporting of MRS studies will help to ensure that MRS studies can be adequately reviewed to standards accepted by the specialist MRS community.

This lack of consistency in reporting was highlighted in a recent meta‐analysis of MRS studies in chronic pain,^5^ leading those authors to propose a minimum quality assessment guide, MRS‐Q.^5^ To this end, an expanded set of guidelines for minimum and recommended reporting requirements is presented in this paper. The origin of these guidelines was a panel at the 2016 International Society of Magnetic Resonance in Medicine (ISMRM) workshop “MR spectroscopy: from current best practice to latest frontiers.” These minimum and recommended requirements were then reviewed and amended by authors selected from the ISMRM Magnetic Resonance Spectroscopy Study Group who have reviewed at least 10 MRS‐focused papers for the following journals: *Magnetic Resonance in Medicine*, *NMR in Biomedicine*, *Journal of Magnetic Resonance*, *Radiology*, and *Magnetic Resonance Materials in Physics, Biology, and Medicine*. These are well‐established peer‐reviewed specialist journals that have focused on MRS‐related topics, which ensures that the authors are considered experts in the technical aspects of MRS and experienced in its scientific use. Recognizing the need to include input from less experienced authors, we also included two trainees as authors to review and edit the manuscript to ensure it was clear to authors new to the field. We then formed the *Experts Working Group on reporting standards for MRS*, who support the paper's recommendations with collaborators with more than 5 years of experience in MRS methodology and application, who either have extended years of service as reviewers for the main MRS journals or are editors of those journals. This follows the same pathway to consensus as the other consensus papers in this special issue.^6^, ^7^, ^8^, ^9^, ^10^, ^11^, ^12^, ^13^, ^14^, ^15^, ^16^, ^17^ These consensus papers provide context to these recommendations, and for further details, as indicated throughout the paper, new authors should reference these papers.

In order to facilitate implementation of these guidelines, a checklist of minimum requirements for the publication of MRS studies was developed (Table 1)—the Minimum Reporting Standards for in vivo Magnetic Resonance Spectroscopy (MRSinMRS) checklist—and exemplar filled in versions are included as appendices (Appendix 1, Appendix 2, Appendix 3, and Appendix 4). The intention is that, for papers utilizing MRS, the authors would complete the table and submit it to the journal, in addition to their manuscript, for review, or use the table to check whether all essential parameters have been listed in the Methods part of the manuscript, with the table subsequently to be included as an appendix to the article. For single site or nucleus studies the first column should be used, and for multisite or multisequence studies it is recommended to complete additional columns as appropriate. Likewise, in the appendices of this paper, several examples have been provided to illustrate how this table should be completed. The model follows checklists such as STARD,^18^ CONSORT,^19^ PRISMA,^20^ and STROBE.^21^ This will enable editors, reviewers, and ultimately readers to be sure of the MRS methodology employed in particular studies, and to ensure that all sufficient details are available to those intending to reproduce or extend the studies or use the results for meta‐analyses. The checklist (Table 1) will also help to standardize the presentation of MRS information and provide journals less familiar with MRS with a systematic way of certifying the methods used.

**TABLE 1:** MRSinMRS checklist. Additional columns are provided for multisite or multisequence studies if necessary

Site (name or number)			
1. Hardware			
a. Field strength [T]			
b. Manufacturer			
c. Model (software version if available)			
d. RF coils: nuclei (transmit/receive), number of channels, type, body part			
e. Additional hardware			

2. Acquisition			
a. Pulse sequence			
b. Volume of interest (VOI) locations			
c. Nominal VOI size [cm^3^, mm^3^]			
d. Repetition time (*T*_R_), echo time (*T*_E_) [ms, s]			
e. Total number of excitations or acquisitions per spectrum In time series for kinetic studies i. Number of averaged spectra (NA) per time point ii. Averaging method (eg block‐wise or moving average) iii. Total number of spectra (acquired/in time series)			
f. Additional sequence parameters (spectral width in Hz, number of spectral points, frequency offsets) If STEAM:, mixing time (*T*_M_) If MRSI: 2D or 3D, FOV in all directions, matrix size, acceleration factors, sampling method			
g. Water suppression method			
h. Shimming method, reference peak, and thresholds for “acceptance of shim” chosen			
i. Triggering or motion correction method (respiratory, peripheral, cardiac triggering, incl. device used and delays)			

3. Data analysis methods and outputs			
a. Analysis software			
b. Processing steps deviating from quoted reference or product			
c. Output measure (eg absolute concentration, institutional units, ratio), processing steps deviating from quoted reference or product			
d. Quantification references and assumptions, fitting model assumptions			

4. Data quality			
a. Reported variables (SNR, linewidth (with reference peaks))			
b. Data exclusion criteria			
c. Quality measures of postprocessing model fitting (eg CRLB, goodness of fit, SD of residual)			
d. Sample spectrum			

## 2 REPORTING GUIDELINES

Below we set out in five sections the important pieces of information about an MRS study that are to be considered as either requirements, or recommendations, along with reasons why these are considered important. A more in‐depth description of terminology and abbreviations to be used can be found in the work of Kreis et al,^11^ while a fuller discussion of several concepts are to be found in other consensus papers in this special issue.^6^, ^7^, ^8^, ^9^, ^10^, ^11^, ^12^, ^13^, ^14^, ^15^, ^16^, ^17^

### 2.1 MRI system description

- Field strength, *eg 1.5 T, 3 T, 7 T, 9.4 T*
- Manufacturer, *eg General Electric, Philips, Siemens, Toshiba*
- Model, eg *General Electric Signa HD/X/T/de, Optima MR450/MR450W, Discovery MR750/MR750W, Signa Premier; Siemens Biograph mMR, Magnetom, Aera, Espree, Prisma, Skyra, Trio, Verio, Magnetom 7 T, Terra; Phillips Ingenia 1.5 T S, 3 T X/S, Elition 3 T X/S, Ambition 1.5 T X/S, Achieva 1.5 T/3 T*. Software version, *eg Siemens VB17A, VD19, VE11C; General Electric 12x‐24x; Phillips Release 5, 5.1 (R1‐3), 5.6, 6*
- RF coils used (nuclei, number of channels, type, body part), *eg* ^*1*^ *H,* ^*31*^ *P,* ^*13*^ *C,* ^*31*^ *P‐* ^*1*^ *H; type, eg head/neck, torso, knee; if not manufacturer, design, eg butterfly, quadrature etc*
- Additional hardware, *eg shim inserts, dielectric pads*

Rationale:

Full and accurate description of the MR system and MR hardware allows for appropriate comparison across studies.

- ***Field strength.*** First and foremost is the field strength of the MR system utilized, expressed in tesla. This allows a reader to position the results in the wider literature. Field strength also has implications for the sensitivity of the MRS approaches employed, and for problems or issues that may exist^2^, ^14^, ^17^ (eg increased chemical shift displacement error with higher field strength, differences in spectral dispersion and hence appearance). Specifying the exact resonance frequency in megahertz in addition to the field strength may be useful, in particular for meta‐analyses and data sharing purposes, because the field strength is usually indicated with zero‐ or single‐digit precision only. Indicating the resonance frequency is particularly encouraged where the actual field strength deviates considerably from the rounded value indicated for B_0_ (eg 123.06 MHz with 2.89 T instead of 127.74 MHz at 3.0 T).
- ***Manufacturer***. While the physical principles governing MRS are well understood, different vendor approaches can lead to systematic differences in results, and therefore manufacturer information should be included.
- ***Model***. Hardware differences exist depending on the model of the scanner, for example the bore size and gradient hardware, which impact on *B*_0_ homogeneity and echo‐planar spectroscopic imaging performance, respectively.^22^ The *software version* is often omitted, but should also be given whenever possible, as some special features such as frequency correction and shimming algorithms may differ between different software versions.
- ***RF coils***. The RF coil information should include the nuclei the coil is tuned to so that it is clear which nuclei are observed. For double‐tuned coils, both nuclei should be indicated with a forward slash in between. As the coil design can have a major impact on the data acquired, it is important to include all the relevant details of the coil such as whether a single coil was used for transmit and receive and/or the number of channels for phased array receive and transmit coils. If it is not a standard manufacturer product coil, further details such as the design of the coil should be included and a reference for a previous publication that may provide more detail.
- ***Additional hardware***. Finally, details should be included of any additional hardware used, such as shim/gradient inserts, dielectric pads, or any other modification of the hardware used for data acquisition.

### 2.2 Acquisition parameters in full

- Pulse sequence, *eg spin‐echo, point resolved spectroscopy (PRESS), stimulated echo acquisition mode (STEAM), semi‐LASER, etc*.
- Location of volume(s) of interest (VOI(s)), *eg posterior cingulate gyrus, M. tibialis anterior, internal capsule of prostate, etc*. A figure that displays the VOI on anatomic images is recommended.
- Nominal VOI size [cm^3^, mm^3^], *eg 40 × 40 × 10 mm* ^*3*^.
- Repetition time (*T*_R_), echo time (*T*_E_) [ms, s]; if STEAM, mixing time (*T*_M_).
- Total number of excitations per spectrum.
- Additional sequence parameters, eg

- Spectral width [Hz, kHz] and number of data points
- Frequency offset (if any)
- If magnetic resonance spectroscopic imaging (MRSI): specification of two‐dimensional (2D) or three‐dimensional (3D) spatial mapping, field of view (FOV), matrix size, acceleration factor, sampling/reconstruction method (eg parallel imaging, compressed sensing, spatial spectral encoding, etc), nominal and effective (ie final) voxel volumes, flip angles for fast MRSI
- For multidimensional acquisitions, number of encodings in the second spectral dimension
- For editing methods, editing pulse information including pulse shape, bandwidth and offset frequency
- For multinuclear sequences: details of decoupling or polarization transfer sequences and related parameters.

- Water suppression method (and any other suppression methods used, eg lipid suppression, outer volume suppression).
- Shimming method,^10^ reference peak used for assessing shim performance, and thresholds for “acceptance of shim” chosen.
- Triggering method, if used (respiratory, peripheral, cardiac triggering, including device used and delays).
- Frequency and motion correction methods, if used (prospective or retrospective, external tracker or navigator method).

**FIGURE 1:**
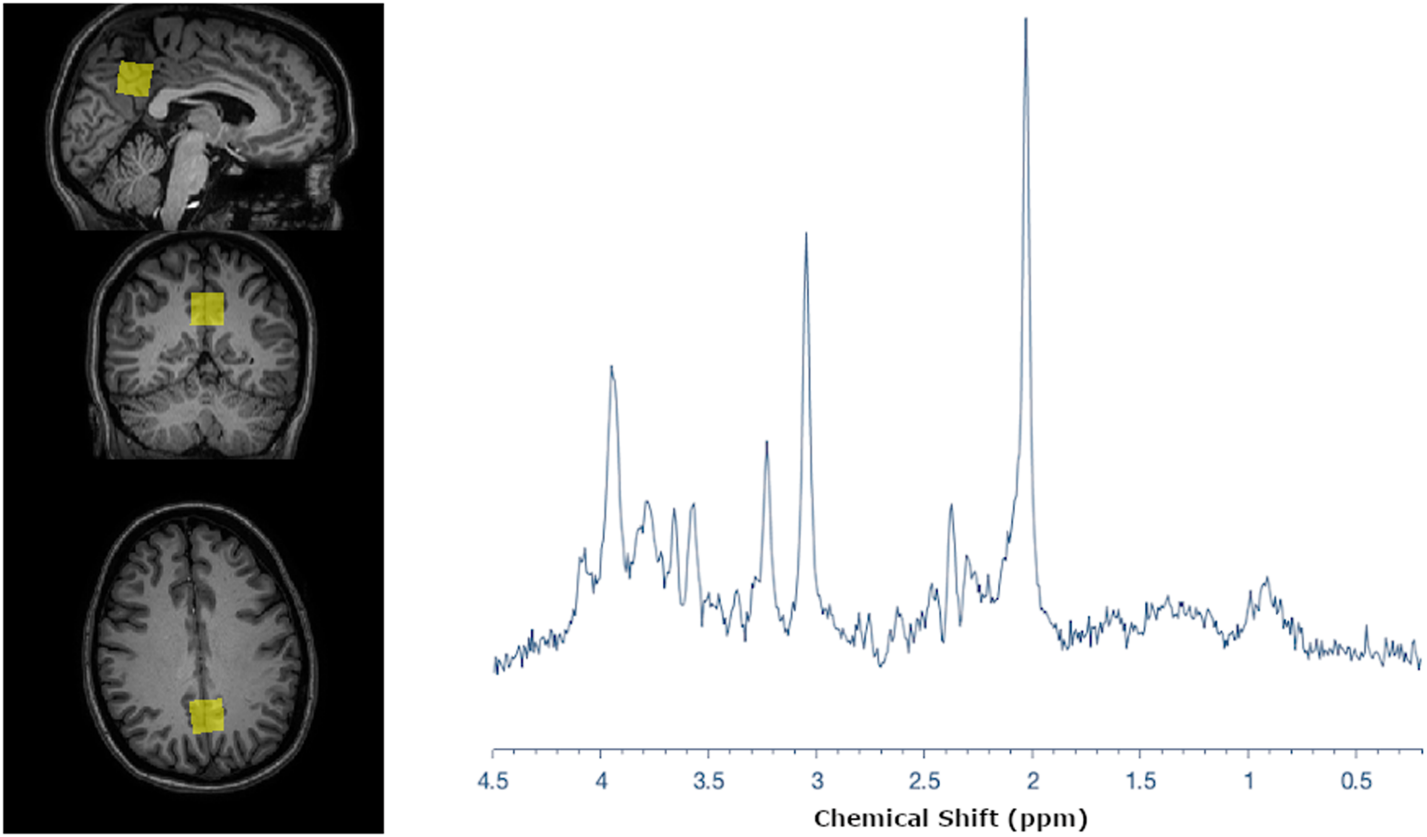
Representative spectrum and voxel location. Representative PRESS spectrum from the posterior cingulate acquired on a 3 T Philips Ingenia. In this figure the raw data from one participant are shown; however, mean data with SDs, multiple data sets, and fitted data may also be shown, as long as the raw data are presented in a fashion that allows an assessment of data quality. The chemical shift axis is labeled in ppm units. Data were collected in accordance with the WMA Declaration of Helsinki

Rationale:

- ***Pulse sequence***. The pulse sequence dictates the parameters that need to be described under ***additional sequence parameters*** **.** Citing the original article that first introduced and described this sequence in detail is recommended along with outlining important deviations from the original sequence, and if the sequence is vendor supplied or a customized sequence.
- ***Location*** **.** The voxel location is the anatomical position of the VOI selected for single‐voxel spectroscopy or the excitation or selection volume in MRSI methods. It should be described in the checklist table in brief and in the manuscript be either shown in a figure or described in detail with anatomical landmarks. It is important to address concerns regarding regional specificity of results and possible tissue‐specific effects (ie for brain gray matter, white matter, and cerebrospinal fluid content).
- *
**VOI** **size***. The VOI size must include the dimensions along the right‐left, anterior‐posterior, and superior‐inferior directions with anatomical referencing if relevant. For MRSI, this should be the excitation volume. Regional analyses for MRSI can use signal averaging over multiple voxels, which should also be described in detail if used.
  It is important to show ***example spectra*** obtained from these regions to allow the reviewer and reader to assess the quality of the data. The spectra should be representative, and, if possible, visualize the studied effect by comparison with a reference spectrum (eg healthy subject/tissue versus patient/affected tissue; physiological conditions such as rest versus end of exercise for muscle). See also the quality assurance section. In recognition of the limited space for figures in some journals, a figure containing the VOI and corresponding MR spectra could be placed in the appendix or supplemental data. See Figure 1 for an example.
- *T**iming parameters*** **(*echo time* and *repetition time*)** are considered essential parameters as these will affect the way spectra appear. ***T***_**E**_ and ***T***_**R**_ lead to differential *T*_2_ and *T*_1_ relaxation effects, with this effect being present between different metabolites. The importance of this is best illustrated by considering total creatine (tCr) and total choline (tCho). The methyl signal of tCr is a commonly utilized internal reference peak; however, its *T*_2_ relaxation constant is shorter than that for tCho.^23^, ^24^ This means that for studies with long *T*_E_ (eg 144 ms) the tCho/tCr peak height or area ratio will be larger than for studies with shorter *T*_E_ (eg 30 ms). This can lead to a misinterpretation of differences between two different studies if the *T*_2_ relaxation difference is not considered. For STEAM sequences ***mixing time*** (***T*** **M**; the time between the second and third 90° RF pulses) will affect the evolution of multiquantum coherence,^25^ and so may impact quantification even if the effective *T*_E_ is the same between two studies. Similar effects can be seen for changes in standard vendor‐implemented sequences' timings for other acquisition schemes, and so information on any such changes should always be provided.
- ***Number of excitations/acquisitions***. The signal‐to‐noise ratio (SNR) in MRS is dependent on VOI size and the ***number of acquisitions*** (NA; averages or phase encodings in MRSI). More acquisitions lead to an improved SNR, which in turn improves reliability of fitting. Different approaches to data acquisition between vendors and groups mean that MRS data may be acquired as either an average or sum of multiple acquisitions, or as an average of a series of blocks, which themselves contain a set number of acquisitions, or number of excitations. Describing both the number of acquisitions per block/scan, and if present the number of blocks, allows the total number of acquisitions for the entire acquisition to be calculated. It is recommended that this total number of scans per acquisition/analysis be reported. This is of particular importance for kinetic studies, in which the data are acquired as time series. Here the number of excitations per time point, signal averaging method (eg block‐wise or moving average), and total number of spectra (acquired/in time series) should be reported (see the consensus paper by Meyerspeer et al^15^ in this special issue for details of reporting on kinetic studies).
- ***Additional sequence parameters***. These will be determined by the sequence used for acquisition. For most methods, however, it would be appropriate to describe the spectral width in hertz and the number of data points acquired. If any frequency offset is used, it should also be described.
  For MRSI methods, the necessary details include the FOV and matrix size so that the nominal volume of MRS voxels can be determined. Acceleration methods (such as parallel imaging, compressed sensing, or spatial‐spectral encoding) can be used to reduce the scan times required for MRSI methods and should be described with details of the method and parameters used.^26^ Similarly, *k*‐space weighting of the acquisition should also be described, such as whether full or elliptical *k*‐space sampling is used or retrospective filters used (eg Hamming), and any *k*‐space zero‐filling factors applied. These factors will impact the effective, ie resultant, voxel volume, which should also be stated if known.^14^
  Additional modifications to the default settings of the pulse sequence should be described. For example, if a frequency offset for excitation of water‐suppressed scans is used to address chemical shift differences between the water reference and metabolite scans, this should be specified, either as offset frequency from water or as chemical shift value in parts per million (ppm) on the standard MRS frequency axis. Reporting of sub‐echo times (*T*_E1_, *T*_E2_), if known, is also recommended.
  Edited MRS sequences require reporting of several more advanced parameters, for example the bandwidth and frequency of editing pulses used, co‐edited metabolites, and specific timing parameters or acquisition schemes. (For more detail and recommendations on spectral editing, see the consensus paper by Choi et al^8^ in this special issue.) Similarly, for multinuclear sequences, details of decoupling, editing, or polarization transfer sequences have to be indicated and related parameters provided.
- ***Water and fat suppression***. Water suppression is a key element of the data acquisition in proton MRS (^1^H MRS), as both the method used, and the degree of water suppression, can greatly influence the spectral quality and analysis of the data. The type of water suppression used should be specified if specific water suppression methods are selected. If the authors used the default water suppression method for their choice of pulse sequence, it is acceptable to report “Standard,” as manufacturers often do not specify which water suppression method is used. If there are parameters related to water suppression such as “weak water suppression” as specified on Siemens systems, or the bandwidth of the water suppression pulses, this should be listed. For further details see the consensus paper by Tkáč et al^6^ in this special issue. As with water, fat suppression techniques may also impact data quality, and if used, specifics should also be listed (eg frequency offset, number and location of outer voxel suppression bands, bandwidth).
- ***Shimming method***. Similarly, different shimming methods may be selected at the time of acquisition. In most cases, authors will utilize the vendor‐provided automated shimming, which usually involves the use of a gradient echo field map to optimize the *B*_0_ field homogeneity, but may employ other methods (eg “pencil beam” VOI in Philips). If a vendor‐supplied methodology is used, the authors should state this; if first, second, or third order shims are used; and describe whether or not the resulting linewidth was measured and used for quality assurance. Ideally, studies should measure the linewidth (to be reported as full‐width at half‐maximum, FWHM) for the unsuppressed water resonance or a specific metabolite peak^11^ in each examination and report the threshold at which shimming was considered acceptably achieved, and how this was assessed (eg system reported results for shim, phase or magnitude spectrum, or other). If manual shimming is used this fact should be listed in the checklist table along with the maximum linewidth allowed. More details on shimming are described in the consensus paper by Juchem et al^10^ in this special issue.
- ***Triggering method***. If used it should be mentioned. This can then be considered, along with *T*_R_, to ascertain if any *T*_1_ effects are likely to have an impact on data and SNR. For example, triggering via cardiac measures might cause a shorter *T*_R_ in exercise studies during a period of exercise that increases heart rate.
- ***Frequency and motion correction***. Methods available vary, with the possibility of retrospective or prospective correction for frequency shifts caused by field drift, motion, or other factors. Reporting the methodology used, and at what part of the process it occurred, allows for more accurate replication of future studies, as well as appropriate comparisons between studies. For more details, see the consensus paper on frequency and motion correction by Andronesi et al^7^ in this special issue.

### 2.3 Spectral quantification methods and parameters

- Software package used to reconstruct and analyze the MRS data including MR manufacture software (eg *General Electric PROBE, Siemens Syngo, or Phillips SpectroView*) and/or third‐party software packages (eg *LCModel,* ^27^ *jMRUI,* ^28^ *TARQUIN,* ^29^ *SIVIC,* ^30^ *INSPECTOR,* ^31^ *FID‐A,* ^32^ *BrainSpec,* ^33^ *MIDAS,* ^34^ *GANNET* ^35^)
- Deviations in processing steps from quoted reference or product defaults
- Quantitative output measures
- Quantification references and assumptions, model fitting assumptions

Rationale:

- ***Software package***. The software packages used to reconstruct and analyze the MRS data including MR manufacturer software and/or third‐party software packages must be described in the table under the ***Analysis software*** section. If the authors used vendor‐provided software this should be specified, or if third‐party software is used it should be described in the table, and a suitable reference provided. Different analysis packages have different approaches to parameter estimation, which may impact the results.^16^ If more than one software package was used, they should all be listed along with the aspects of the analysis for which they were used.
- ***Deviations in processing steps***. Any automatic and manual processing steps deviating from a software package’s default analysis have to be listed: for example, changes to phasing, frequency alignment, eddy current corrections. For phased array coils, any alterations to coil combination should be described. In addition, it should be described if these changes are performed on single acquisitions before averaging. References should be provided that describe the methodology and its specifics rather than publications that simply utilize the method.
- ***Quantitative output measures***. The output measure of the spectral analysis should be described. There are three main ways that MRS metabolite concentrations can be described.
  First, MRS results are often reported as a ratio of the primary metabolite to another. This can be done using the ratio of the peak area measurements, or ratio of relative concentrations, which accounts for the number of resonant nuclei in each compound. It is important to indicate which metabolite is used as the denominator.
  The second method is to report the metabolites as “institutional units,” which is the signal reported by the software, normalized such that measures at different time points or from different subjects can be compared. This normalization usually stops short of all steps required to report conventional concentration estimates. The most basic measure is based on the peak height of the metabolite, but this is greatly influenced by the linewidth, and therefore reporting the area under the curve (or the equivalent measure for time‐domain fitting) is recommended. In either case the baseline fitting method should be described. A common approach for normalization to institutional units in ^1^H MRS is to take the ratio of the fitted metabolite signal to the fit of the unsuppressed water resonance.
  Finally, metabolite concentrations can be expressed in “absolute units” (standard chemical units, such as millimoles per wet weight, molar, or molal) using some conversion methods, which usually rely on multiple assumptions (eg an assumed tissue content for the reference component). In order to provide estimates of metabolite concentration, contributions to the signal from different tissue compartments should be considered. For the brain this means that gray matter, white matter, and cerebrospinal fluid volumes calculated via voxel segmentation should be reported as appropriate, especially if water is used as the internal reference. If relaxation correction is applied, listing *T*_2_ and *T*_1_ values used (and/or a suitable reference) is necessary.
- ***Quantification references and assumptions, and model fitting***. Some software packages utilize model‐fitting methods for spectral analysis. In those cases, the models used should be described in detail, as the number of metabolites used can greatly impact the result; for example, were the models simulated, and if so using what software (eg VESPA,^36^ GAMMA,^37^ FID‐A,^32^ NMR‐PROBE or NMRSCOPE in jMRUI,^28^ MARRS, etc). The basis set used should be described either as the “default” basis set provided with the software, or if it was modified which metabolites were included in the basis set. In addition, the fitting model also has to be specified in terms of implemented parameter relations and constraints. This must be spelled out in full if deviating from default parameter sets for the specific versions of the fit packages or quoted literature reference.
  Moreover, for brain ^1^H MRS spectra information on how the macromolecule signals were handled in the fitting procedure is mandatory. This can be done either by using a spectrum of macromolecules acquired in vivo, or by a mathematical approach, which is usually incorporated in the software package. When the mathematical approach is used, details of how it was done also need to be mentioned, ie for QUEST in jMRUI the number of points used or for LCModel number of macromolecules and lipid peaks included. For more detail on macromolecule contributions in MRS see the consensus paper by Cudalbu et al^9^ in this special issue.

### 2.4 Quality assurance. Studies must include the following

- Reported variables (SNR, linewidth, and description of how they were obtained)
- Data exclusion criteria
- Other quality measures from fitting software are also recommended (eg standard deviation (SD), Cramér‐Rao lower bound (CRLB)), and/or the robustness of the measures gained (repeatability measures if known)
- Figure showing representative spectra

Rationale:

- ***Reported variables***. One of the greatest challenges of the MRS literature is the evaluation of spectral quality. There are no agreed standards for data quality. While no single measure is the gold standard of data quality, the primary measures in practice are SNR, spectral linewidth, and CRLB. As SNR can be measured in many different ways, it is important that authors report both the SNR and its measurement method (see the work of Kreis et al^11^ and Oz et al^1^).
***Linewidths*** are typically measured as the FWHM of the fitted resonance. For ^1^H MRS, this may be done using the water resonance and determined either at the time of acquisition during the pre‐scan shimming routine or post hoc through a spectral analysis of the water spectrum. These values should be reported to ensure that spectra are of adequate quality to analyze. In non‐^1^H MRS, usually the most prominent singlet resonance in the spectrum is used to measure linewidth (eg PCr). Linewidths can also be obtained from the output of fitting packages, where they would usually indicate the linewidth of specific metabolite signals. It is important to specify the origin of the linewidth indicated.
- ***Data exclusion criteria***. The data exclusion criteria should specifically provide the thresholds for which data were excluded, whether they were based on SNR, linewidth, and/or other quality measures, and the specifics of this measure, as this can bias the overall analysis of the study data.^18^ For example, “subjects were excluded if the SNR of tCr was less than 5 or the FWHM was greater than 12 Hz.” Note that, to avoid bias,^38^ if CRLBs are used as exclusion criteria they should not be in the form of percentage values of a metabolite of interest that can have a small value in an individual subject, but rather be formulated in absolute concentration units (or relative to a stable reference metabolite). It is also important to describe how many subjects or voxels per subject cohort were eliminated based on the specified criteria.
- ***Quality measures of model fitting***. Additional measures of goodness of fit, or fit error, should be reported where applicable (eg CRLB for lower bound of the fit error, or SD). If reproducibility or repeatability^11^ of a measure has been shown, it is recommended to report it to demonstrate the robustness of single measures.
- ***Representative spectrum***. Finally, one of the most important methods of quality control is visual inspection of the MR spectrum by experienced users or MRS experts (note: in MRSI visual inspection of metabolic maps becomes equally important^14^). Sample spectra are required so that both reviewers and readers can assess the quality and interpretation of the MRS data (see the ‘Acquisition’ section in the selection criteria for representative spectra). While a single spectrum may of course not reflect the quality of all of the data, it does provide a general assessment. In contrast, selecting a single spectrum from thousands of spectra in MRSI may not reflect the overall MRSI data quality, and so maps, with details of how they are scaled and exemplar spectra displayed, are more appropriate. The requirements for this spectrum include the following. (1) The raw spectrum must be shown, not the fitted data alone, as the fitted data do not reflect the SNR or potential systematic artifacts. If the baseline is calculated, it is recommended to show it. Fitted data are recommended to be shown in addition as an overlay to reflect quality of model fitting. (2) The *x* axis or chemical shift axis should be displayed with units in ppm. (3) If the spectrum is apodized for display purposes, the apodization parameters should be given in the figure caption. An example is shown in Figure 1. As described above, spectra can be included in a figure that also presents the voxel location to meet space and figure constraints for specific journals. In addition, for a more complete representation of spectral quality in the study, plotting of the average spectrum across all data points and SDs around this average from each studied cohort may be displayed. In the checklist, the figure number should be described so that it can be easily referenced and also serves to indicate its presence.

The details described above should be included in the text of the manuscript or in a supplemental methods section. The MRSinMRS checklist (Table 1) is intended to be a reference for the author and the reviewer as well as the reader, and while it should be included as supplemental material it is not intended to replace the manuscript text. Example checklists based on information from existing publications are provided in the appendices of this paper (Appendix 1, Appendix 2, Appendix 3, Appendix 4) to provide guidance to authors as to the details that should be included in the checklist. Those items in the checklist that are in italics are details that should have been but were not included in these publications, illustrating further the value of including the checklist to ensure that all important details are included in the manuscript.

## 3 CONCLUSION

These minimum reporting guidelines for MRS should allow the field to improve the rigor and critical examination of reported results, improve the reproducibility and comparability of studies, and provide new entrants to the field with detailed guidance as to reporting practices. To assist authors in reporting and reviewers in assessing these essential and recommended parameters, we have provided a simplified checklist (Table 1). It is hoped that this checklist will facilitate writing for authors, improve analysis for journal reviewers, and provide an easy way for journal editors to ensure that MRS studies are reported in full. In addition, reporting requirements, if checked early, encourage researchers to consider these aspects ahead of time, hopefully before data collection has commenced. While it is preferred that details of the MRS acquisition and analysis are included in the main text, the MRS reporting checklist can also be provided as part of the appendix or supplementary material of a submission and used in the review process, as with many other manuscript checklists such as PRISMA, STARD, CONSORT, and STROBE. (Researchers may also find the MRS‐Q V1 form at Open Science Framework (https://osf.io/8s7j9/) useful.)

Adherence to these minimum requirements and recommended guidelines is expected to ensure that all MRS papers provide the necessary information to reproduce studies as well as provide a basis for comparison for the evaluation of the studies across clinical domains. As with initiatives in other fields of biological and clinical research, it is expected that this will improve reproducibility and validity, and strengthen the field going forward.

### EXPERTS' WORKING GROUP ON REPORTING STANDARDS FOR MR SPECTROSCOPY

Gareth J Barker, Professor of Magnetic Resonance Physics, King's College London, Institute of Psychiatry, Psychology & Neuroscience, Box 089, DeCrespigny Park, London, SE5 8AF, UK.

Brenda Bartnik Olson, PhD ‐ Physicist , Associate Professor Loma Linda University Health, Department of Radiology, California, USA.

William M. Brooks, PhD Director, Hoglund Biomedical Imaging Center Associate Director, Frontiers: KU Clinical & Translational Science Institute Professor, Department of Neurology University of Kansas Medical Center 3901 Rainbow Blvd Kansas City, KS 66160.

Chuck Gasparovic The Mind Research Network Albuquerque NM.

Ashley D. Harris, Department of Radiology, University of Calgary, Calgary, Canada.

Franklyn A. Howe, DPhil Professor of Magnetic Resonance Imaging Head of the Neurosciences Research Centre St George's, University of London Cranmer Terrace, London, SW17 0RE, UK.

Ivan I. Kirov, PhD, Assistant Professor, Department of Radiology NYU Langone Health, New York, USA.

Bernard Lanz, Laboratory for Functional and Metabolic Imaging (LIFMET), Ecole Polytechnique Fédérale de Lausanne, Lausanne, Switzerland.

Mary McLean Department of Radiology, University of Cambridge School of Clinical Medicine, Cambridge, UK.

Ralph Noeske, PhD Senior Scientist Global Spectroscopy Leader Applied Science Lab Europe GE Healthcare.

Vincent O. Boer Danish Research Centre for Magnetic Resonance, Centre for Functional and Diagnostic Imaging and Research, Copenhagen University Hospital Hvidovre, Hvidovre, Denmark.

Harish Poptani Professor and Chair Centre for Preclinical Imaging Department of Molecular & Clinical Cancer Medicine Institute of Systems, Molecular & Integrative Biology Nuffield Wing, Sherrington Building Crown Street Liverpool L69 3BX UK.

Dr Chris Rodgers, Wolfson Brain Imaging Centre, University of Cambridge.

Laura M. Rowland Department of Psychiatry Maryland Psychiatric Research Center University of Maryland Baltimore.

Brian J. Soher, Associate Professor of Radiology, Duke University Medical Center, Durham, NC 27710 USA.

Sunitha B. Thakur, PhD, Departments of Medical Physics and Radiology, Memorial Sloane Kettering Cancer Center, USA.

Ruth O'Gorman Tuura, PD Dr. phil. Head, Center for MR Research University Children's Hospital Zurich Eleonore Foundation Steinwiesstrasse 75 CH‐8032 Zürich.

Julien Valette Atomic Energy and Alternative Energies Commission|CEA, Molecular Imaging Research Center (MIRCen).

Martin Wilson Centre for Human Brain Health and School of Psychology, University of Birmingham, Birmingham, UK.

## Supporting information


**Table S1.** MRSinMRS checklist. Additional columns are provided for multi‐site or multi‐sequence studies if necessary.
**Appendix S1**: Example of the MRSinMRS checklist for a single voxel ^1^H‐MRS study
**Appendix S2**. Example of the MRSinMRS checklist for a multi‐sequence multi‐nuclear MRS study (^1^H, ^31^P)
**Appendix S3**: Example of the MRSinMRS checklist for an X‐nuclear MRS study (dynamic ^31^P MRS, muscle)
**Appendix S4**. Example of the multi‐sequence MRSinMRS checklist for a combined single‐voxel and magnetic resonance spectroscopic imaging study.

## Data Availability

Data sharing is not applicable to this article as no new data were created or analyzed in this study.

## APPENDIX 1

### EXAMPLE OF THE MRSinMRS CHECKLIST FOR A SINGLE VOXEL ^**1**^H‐MRS STUDY

**Table:**

1. Hardware	
a. Field strength [T]	3 T
b. Manufacturer	Siemens
c. Model (software version if available)	Verio (VB17)
d. RF coils: nuclei (transmit/receive), number of channels, type, body part	32 channel head coil
e. Additional hardware	N/A

2. Acquisition	
a. Pulse sequence	3D localized correlated spectroscopy
b. Volume of interest (VOI) locations	Posterior cingulate gyrus
c. Nominal VOI size [cm^3^, mm^3^]	3 × 3 × 3 cm^3^
d. Repetition time (*T*_R_), echo time (*T*_E_) [ms, s]	*T*_R_ 1500 ms, *initial T*_*E*_ *30 ms*, 0.8 ms increments
e. Total number of excitations or acquisitions per spectrum In time series for kinetic studies i. Number of averaged spectra (NA) per time point ii. Averaging method (eg block‐wise or moving average) iii. Total number of spectra (acquired/in time series)	64 increments with 8 averages per increment
f. Additional sequence parameters (spectral width in Hz, number of spectral points, frequency offsets) If STEAM: mixing time (*T*_M_) If MRSI: 2D or 3D, FOV in all directions, matrix size, acceleration factors, sampling method	F1/F2: 2000 Hz/1250 Hz, 1024 points
g. Water suppression method	WET
h. Shimming method, reference peak, and thresholds for “acceptance of shim” chosen	Automated *B*_0_ field mapping followed by manual shimming of water to <14 Hz
i. Triggering or motion correction method (respiratory, peripheral, cardiac triggering, incl. device used and delays)	N/A

3. Data analysis methods and outputs	
a. Analysis software	Felix‐2007
b. Processing steps deviating from quoted reference or product	F2 domain (skewed sine‐squared window, 2048 points, magnitude mode), F1 domain (sine‐squared window, linear prediction to 96 points, zero‐filling to 512 points, magnitude mode)
c. Output measure (eg absolute concentration, institutional units, ratio)	Ratio to creatine
d. Quantification references and assumptions, fitting model assumptions	Each spectrum was calibrated by setting the lysine cross peak (at 3.00‐1.67 ppm) and specifying a constant ‘number of contour levels’ (set to 28), as well as a constant ‘level multiplier’ (defined as the difference between values of consecutive contour, set to 1.05).

4. Data quality	
a. Reported variables (SNR, linewidth (with reference peaks))	*SNR and linewidth not described*
b. Data exclusion criteria	*No subjects excluded*
c. Quality measures of postprocessing model fitting (eg CRLB, goodness of fit, SD of residual)	*No QA measures described*
d. Sample spectrum	Figure 1

The example above used the following paper: Lin AP, Ramadan S, Stern RA, et al. Changes in the neurochemistry of athletes with repetitive brain trauma: preliminary results using localized correlated spectroscopy. *Alzheimers Res Ther*. 2015;7(1):13. Items listed in italics are details that were not included in the paper that served as the source for this example.

The example above used the following paper: Lin AP, Ramadan S, Stern RA, et al. Changes in the neurochemistry of athletes with repetitive brain trauma: preliminary results using localized correlated spectroscopy. *Alzheimers Res Ther*. 2015;7(1):13.
Items listed in italics are details that were not included in the paper that served as the source for this example.

## APPENDIX 2

### EXAMPLE OF THE MRSinMRS CHECKLIST FOR A MULTI‐SEQUENCE MULTI‐NUCLEAR MRS STUDY (^**1**^H, ^**31**^P)

**Table:**

Site (name or number)			
1. Hardware			
a. Field strength [T]	3 T	3 T	
b. Manufacturer	Siemens	Siemens	
c. Model (software version if available)	Skyra (VD13)	Skyra (VD13)	
d. RF coils: nuclei (transmit/receive), number of channels, type, body part	32 channel ^1^H head coil	*10 cm* ^31^P tuned transmit/receive surface coil	
e. Additional hardware	N/A	Custom‐built dynamic knee extension apparatus	

2. Acquisition			
a. Pulse sequence	PRESS	500 μs pulse and acquire	
b. Volume of interest (VOI) locations	Posterior cingulate gyrus	N/A	
c. Nominal VOI size [cm^3^, mm^3^]	30 × 30 × 30 mm^3^	N/A	
d. Repetition time (*T*_R_), echo time (*T*_E_) [ms, s]	*T*_R_/*T*_E_ = 2000/30 ms	*T*_R_ = 2000 ms	
e. Total number of excitations or acquisitions per spectrum In time series for kinetic studies i. Number of averaged spectra (NA) per time point ii. Averaging method (eg block‐wise or moving average) iii. Total number of spectra (acquired/in time series)	64 averages	300 total FIDs SIFT used for averaging	
f. Additional sequence parameters (spectral width in Hz, number of spectral points, frequency offsets). If STEAM: mixing time (*T*_M_). If MRSI: 2D or 3D, FOV in all directions, matrix size, acceleration factors, sampling method	1200 Hz, 1024 data points	3000 Hz, 2048 data points	
g. Water suppression method	CHESS	N/A	
h. Shimming method, reference peak, and thresholds for “acceptance of shim” chosen	Automated *B*_0_ field mapping followed by manual shimming of water to <14 Hz	Automated *B*_0_ field mapping	
i. Triggering or motion correction method (respiratory, peripheral, cardiac triggering, incl. device used and delays)	None	None	

3. Data analysis methods and outputs			
a. Analysis software	LCmodel vers 6.2	jMRUI	
b. Processing steps deviating from quoted reference or product	None	SIFT pre‐processing prior to use of jMRUI	
c. Output measure (eg absolute concentration, institutional units, ratio), processing steps deviating from quoted reference or product	Ratios to creatine	PCr amplitude	
d. Quantification references and assumptions, fitting model assumptions	Default basis set	AMARES Gaussian lineshapes	

4. Data quality			
a. Reported variables (SNR, linewidth (with reference peaks))	*SNR: 61.8 ± 6 (51‐71) FWHM: 0.043 + 0.006 (0.38‐0.57) ppm as reported by LCmodel* *None eliminated*	*SNR and FWHM not reported*	
b. Data exclusion criteria	*SNR < 40; CRLB > 20%*	*SD > 10%*	
c. Quality measures of postprocessing model fitting (eg CRLB, goodness of fit, SD of residual)	*CRLB of NAA: 2 ± 0(2)%*	*SD: 4.5 ± 1.7%*	
d. Sample spectrum	Figure 1	Figure 2	

The example above used the following paper: Zhou M, Liao H, Sreepada LP, Ladner JR, Balschi JA, Lin AP. Tai chi improves brain metabolism and muscle energetics in older adults. *J Neuroimaging*. 2018;28(4):359‐364. https://doi.org/10.1111/jon.12515. Items listed in italics are details that were not included in the paper that served as the source for this example.

The example above used the following paper: Zhou M, Liao H, Sreepada LP, Ladner JR, Balschi JA, Lin AP. Tai chi improves brain metabolism and muscle energetics in older adults. *J Neuroimaging*. 2018;28(4):359‐364. https://doi.org/10.1111/jon.12515.
Items listed in italics are details that were not included in the paper that served as the source for this example.
NAA, *N*‐acetylaspartate.

## APPENDIX 3

### EXAMPLE OF THE MRSinMRS CHECKLIST FOR AN X‐NUCLEAR MRS STUDY (DYNAMIC ^**31**^P MRS, MUSCLE)

**Table:**

1. Hardware	
a. Field strength [T]	7 T
b. Manufacturer	Siemens Healthineers, Erlangen, Germany
c. Model (software version if available)	Magnetom 7 T (VB17)
d. RF coils: nuclei (transmit/receive), number of channels, type, body part	Custom‐built three channel ^31^P (*d* = 15 cm, *l* = 10 cm), two channel ^1^H (*d* = 17 cm, *l* = 12.5 cm) transceiver coil, shaped to the human calf (Goluch et al. *Magn Reson Med*. 2015;73(6):1190‐1195)
e. Additional hardware	Custom‐built pedal ergometer with pneumatic piston and MR‐compatible sensors for pedal angle and force

2. Acquisition	
a. Pulse sequence	Semi‐LASER
b. Volume of interest and VOI locations	Single voxel placed obliquely in gastrocnemius muscle, avoiding subcutaneous fat, fasciae and adjacent muscles
c. Nominal VOI size [cm^3^, mm^3^]	Anatomy matched, 27 ± 6 cm^3^ *(ca. 2 × 3.5 × 4 cm* ^*3*^ *)*
d. Repetition time (*T*_R_), echo time (*T*_E_) [ms, s]	*T*_R_ = 6 s, *T*_E_ = 29 ms
e. Total number of excitations or acquisitions per spectrum (NA) In time series for kinetic studies i. Number of averaged spectra) per time point (NA) ii. Averaging method (eg block‐wise or moving average) Total number of spectra (acquired/in time series)	*1 acquisition per spectrum (NA = 1), except for pH quantification 90 s after exercise, where NA = 4, with block‐wise averaging* *Total number of spectra in time series was 140, at NA = 1 (or series of 35 spectra at NA = 4)*
f. Additional sequence parameters (spectral width in Hz, number of spectral points, frequency offsets) i. If STEAM: mixing time (*T*_M_) ii. If MRSI: 2D or 3D, FOV in all directions, matrix size, acceleration factors, sampling method	5 kHz, 2048 complex points after removing oversampling
g. Water suppression method	N/A
h. Shimming method, reference peak, and thresholds for “acceptance of shim” chosen	*1st and 2nd order, vendor standard method (DESS sequence in “advanced shim” mode until convergence), line‐width of PCr peak was evaluated post hoc*
i. Triggering or motion correction method (respiratory, peripheral, cardiac triggering, incl. device used and delays)	Subjects were instructed to push the pedal only during times without RF excitation or signal reception*, cued by gradient noise. Adherence to the protocol was inspected via data from the force sensors*.

3. Data analysis methods and outputs	
a. Analysis software	^31^P MR spectroscopy data were processed from raw data exported from the scanner using in‐house developed Python scripts (http://www.python.org) for phasing and channel combination. Signals were phased to the highest peak magnitude of PCr in the frequency domain after 7 Hz Lorentzian apodization and 4 × zero‐filling. The channel combination was then performed by weighted averaging of the raw data (that is, without apodization and zero‐filling). Weights were calculated as proportional to signal, averaged over four resting spectra (excluding the fully relaxed spectrum). Spectra were then fitted in AMARES, as implemented in jMRUI, *version 5.0*
b. Processing steps deviating from quoted reference or product analysis software (vendor, version)	*Gaussian line shapes, soft constraints for frequencies*
c. Output measure (eg absolute concentration, institutional units, ratio) Processing steps deviating from quoted reference or product	Concentrations in institutional units and pH values
d. Quantification references and assumptions, fitting model assumptions	*Quantification relative to total* ^*31*^ *P signal, which was assumed to be constant*. End‐exercise PCr depletion relative to post‐exercise asymptotic value of mono‐exponential fit of recovery

5. Data quality	
a. Reported variables (SNR, linewidth (with reference peaks))	SNR was calculated using the partially saturated resting spectra of each time series by dividing the PCr peak amplitude by the SD of the signal in a region containing only noise, 15 ppm off‐center across 1/16 of the total bandwidth. Linewidths were taken from the AMARES fit of the PCr peak.
b. Data exclusion criteria	*>10% changes of sum of total* ^*31*^ *P signal* *Linewidth of PCr peak > 15 Hz* *Unphysiological pH values (>7.1)* *Splitting of P*_*i*_ *peak*
c. Quality measures of postprocessing model fitting (eg CRLB, goodness of fit, SD of residual)	*SD of residual*
d. Sample spectrum	Figure 2

NAA, *N*‐acetylaspartate. The example above used the following paper: Niess F, Schmid AI, Bogner W, et al. Interleaved ^31^P MRS/^1^H ASL for analysis of metabolic and functional heterogeneity along human lower leg muscles at 7 T. *Magn Reson Med*. 2020;83:1909‐1919. https://doi.org/10.1002/mrm.28088. Items listed in italics are details that were not included in the paper that served as the source for this example.

The example above used the following paper: Niess F, Schmid AI, Bogner W, et al. Interleaved ^31^P MRS/^1^H ASL for analysis of metabolic and functional heterogeneity along human lower leg muscles at 7 T. *Magn Reson Med*. 2020;83:1909‐1919. https://doi.org/10.1002/mrm.28088.
Items listed in italics are details that were not included in the paper that served as the source for this example.

## APPENDIX 4

### EXAMPLE OF THE MULTI‐SEQUENCE MRSinMRS CHECKLIST FOR A COMBINED SINGLE‐VOXEL AND MAGNETIC RESONANCE SPECTROSCOPIC IMAGING STUDY

**Table:**

1. Hardware			
a. Field strength [T]	3 T	3 T	
b. Manufacturer	Siemens	Siemens	
c. Model (software version if available)	Skyra (VD13B)	Skyra (VD13B)	
d. RF coils: nuclei (transmit/receive), number of channels, type, body part	32 ch ^1^H head coil	32 ch ^1^H head coil	
e. Additional hardware	N/A	N/A	

2. Acquisition			
a. Pulse sequence	PRESS	Semi‐LASER CSI	
b. Volume of interest (VOI) locations	Patients: lesion Controls: centrum semiovale	Patients: lesion	
c. Nominal VOI size [cm^3^, mm^3^]	20 × 20 × 20 mm^3^	80 × 80 × 15 mm^3^	
d. Repetition time (*T*_R_), echo time (*T*_E_) [ms, s]	*T*_R_ = 2000 ms, *T*_E_ = 97 ms	*T*_R_ = 1700 ms, *T*_E_ = 97 ms	
e. Total number of excitations or acquisitions per spectrum In time series for kinetic studies i. Number of averaged spectra (NA) per time point ii. Averaging method (eg block‐wise or moving average) iii. Total number of spectra (acquired/in time series)	128 averages	3 averages	
f. Additional sequence parameters (bandwidth in Hz or dwell time in ms, number of spectral points, frequency offsets) If STEAM: mixing time (*T*_M_) If MRSI: 2D or 3D, FOV in all directions, matrix size, acceleration factors, sampling method	1200 Hz, 1024 points	2D: 160 × 160 × 15 mm^3^ FOV; matrix size 16 × 16, no acceleration factor; weighted distribution sampling	
g. Water suppression method	WET	WET	
h. Shimming method, reference peak, and thresholds for “acceptance of shim” chosen	Automated 3D *B*_0_ field mapping technique followed by manual adjustment <14 Hz	Automated 3D *B*_0_ field mapping technique followed by manual adjustment <25 Hz	
i. Triggering or motion correction method (respiratory, peripheral, cardiac triggering, incl. device used and delays)	N/A	N/A	

3. Data analysis methods and outputs			
a. Analysis software	LCmodel 6.2	LCmodel 6.2	
b. Processing steps deviating from quoted reference or product	Custom basis set	Custom basis set	
c. Output measure (eg absolute concentration, institutional units, ratio), processing steps deviating from quoted reference or product	Ratios to creatine	Ratios to creatine	
d. Quantification references and assumptions, fitting model assumptions	*The basis set included spectra of 2HG, NAA, GABA, glutamate, glycine, creatine, myo‐inositol, glutamine, lactate, alanine, acetate, aspartate, ethanolamine, glutathione, phosphorylethanolamine, scyllo‐inositol, taurine, N‐acetylaspartylglutamate, glucose, and choline simulated using real pulses. Macromolecules were not modelled*.	*The basis set included spectra of 2HG, NAA, GABA, glutamate, glycine, creatine, myo‐inositol, glutamine, lactate, alanine, acetate, aspartate, ethanolamine, glutathione, phosphorylethanolamine, scyllo‐inositol, taurine, N‐acetylaspartylglutamate, glucose, and choline simulated using real pulses. Macromolecules were not modelled*.	
4. Data quality			
a. Reported variables (SNR, linewidth (with reference peaks))	*SNR and linewidths not reported*	*SNR and linewidths not reported*	
b. Data exclusion criteria	SNR < 5 or FWHM of creatine peak > 0.143 ppm	75th percentile 2HG/creatine values of the selected voxels	
c. Quality measures of postprocessing model fitting (eg CRLB, goodness of fit, SD of residual)	2HG CRLB < 30%	2HG CRLB < 30%	
d. Sample spectrum	Figures 1‐3	Figures 1‐3	

The example above used the following paper: Zhou M, Zhou Y, Liao H, et al. Diagnostic accuracy of 2‐hydroxyglutarate magnetic resonance spectroscopy in newly diagnosed brain mass and suspected recurrent gliomas. *Neuro‐Oncol*. 2018;20(9):1262‐1271. https://doi.org/10.1093/neuonc/noy022. Items listed in italics are details that were not included in the paper that served as the source for this example.

The example above used the following paper: Zhou M, Zhou Y, Liao H, et al. Diagnostic accuracy of 2‐hydroxyglutarate magnetic resonance spectroscopy in newly diagnosed brain mass and suspected recurrent gliomas. *Neuro‐Oncol*. 2018;20(9):1262‐1271. https://doi.org/10.1093/neuonc/noy022.
Items listed in italics are details that were not included in the paper that served as the source for this example.
