# Supplementary material for: Minimum Reporting Standards for in vivo Magnetic Resonance Spectroscopy (MRSinMRS): Experts' consensus recommendations
Source: NMR Biomed. 2021 Feb 9;34(5):e4484. doi: 10.1002/nbm.4484 (PMC8647919; doi:10.1002/nbm.4484)
Supplement: Supplementary file 1 — Table S1. MRSinMRS checklist. Additional columns are provided for multi‐site or multi‐sequence studies if necessary. Appendix S1: Example of the MRSinMRS checklist for a single voxel 1H‐MRS study Appendix S2. Example of the MRSinMRS checklist for a multi‐sequence multi‐nuclear MRS study (1H, 31P) Appendix S3: Example of the MRSinMRS checklist for an X‐nuclear MRS study (dynamic 31P MRS, muscle) Appendix S4. Example of the multi‐sequence MRSinMRS checklist for a combined single‐voxel and magnetic resonance spectroscopic imaging study. [file NBM-34-e4484-s001.doc]

**Table 1**. MRSinMRS checklist. Additional columns are provided for multi-site or multi-sequence studies if necessary.

| Site (Name or Number) |  |  |  |
| --- | --- | --- | --- |
| 1. Hardware |  |  |  |
| a. Field strength [T] |  |  |  |
| b. Manufacturer |  |  |  |
| c. Model (software version if available) |  |  |  |
| d. RF coils: nuclei (transmit/ receive), number of channels, type, body part |  |  |  |
| e. Additional hardware |  |  |  |
| 2. Acquisition |  |  |  |
| a. Pulse sequence |  |  |  |
| b. Volume of Interest (VOI) locations |  |  |  |
| c. Nominal VOI size [cm^3^, mm^3^] |  |  |  |
| d. Repetition Time (TR), Echo Time (TE) [ms, s] |  |  |  |
| e. Total number of Excitations or acquisitions per spectrum  In time series for kinetic studies   1. Number of Averaged spectra (NA) per time-point 2. Averaging method (e.g. block-wise or moving average) 3. Total number of spectra (acquired / in time-series) |  |  |  |
| f. Additional sequence parameters  (spectral width in Hz, number of spectral points, frequency offsets)  If STEAM:, Mixing Time (TM)  If MRSI: 2D or 3D, FOV in all directions, matrix size, acceleration factors, sampling method |  |  |  |
| g. Water Suppression Method |  |  |  |
| h. Shimming Method, reference peak, and thresholds for “acceptance of shim” chosen |  |  |  |
| i. Triggering or motion correction method  (respiratory, peripheral, cardiac triggering, incl. device used and delays) |  |  |  |
| 3. Data analysis methods and outputs |  |  |  |
| a. Analysis software |  |  |  |
| b. Processing steps deviating from quoted reference or product |  |  |  |
| c. Output measure  (e.g. absolute concentration, institutional units, ratio)Processing steps deviating from quoted reference or product |  |  |  |
| d. Quantification references and assumptions, fitting model assumptions |  |  |  |
| 4. Data Quality |  |  |  |
| a. Reported variables  (SNR, Linewidth (with reference peaks)) |  |  |  |
| b. Data exclusion criteria |  |  |  |
| c. Quality measures of postprocessing Model fitting (e.g. CRLB, goodness of fit, SD of residual) |  |  |  |
| d. Sample Spectrum |  |  |  |

**Appendix 1**: Example of the MRSinMRS checklist for a single voxel ^1^H-MRS study

| 1. Hardware |  |
| --- | --- |
| a. Field strength [T] | 3 T |
| b. Manufacturer | Siemens |
| c. Model (software version if available) | Verio (VB17) |
| d. RF coils: nuclei (transmit/receive), number of channels, type, body part | 32 channel head coil |
| e. Additional hardware | N/A |
| 2. Acquisition |  |
| a. Pulse sequence | 3D localized correlated spectroscopy, |
| b. Volume of Interest (VOI) locations | Posterior cingulate gyrus |
| c. Nominal VOI size [cm^3^, mm^3^] | 3 x 3 x 3 cm^3^ |
| d. Repetition Time (TR), Echo Time (TE) [ms, s] | TR 1500 ms, *initial TE 30 ms*, 0.8 ms increments |
| e. Total number of Excitations or acquisitions per spectrum  In time series for kinetic studies   1. Number of Averaged spectra (NA) per time-point 2. Averaging method (e.g. block-wise or moving average) 3. Total number of spectra (acquired / in time-series) | 64 increment with 8 averages per increment |
| f. Additional sequence parameters (spectral width in Hz, number of spectral points, frequency offsets)  If STEAM:, Mixing Time (TM)  If MRSI: 2D or 3D, FOV in all directions, matrix size, acceleration factors, sampling method | F1/F2: 2000 Hz/1250 Hz, 1024 points |
| g. Water Suppression Method | WET |
| h. Shimming Method, reference peak, and thresholds for “acceptance of shim” chosen | Automated B0 field mapping followed by manual shimming of water to < 14 Hz |
| i. Triggering or motion correction method  (respiratory, peripheral, cardiac triggering, incl. device used and delays) | N/A |
| 3. Data analysis methods and outputs |  |
| a. Analysis software | Felix-2007 |
| b. Processing steps deviating from quoted reference or product | F2 domain (skewed sine-squared window, 2,048 points, magnitude mode), F1 domain (sine-squared window, linear prediction to 96 points, zero-filling to 512 points, magnitude mode). |
| c. Output measure  (e.g. absolute concentration, institutional units, ratio) | Ratio to creatine |
| d. Quantification references and assumptions, fitting model assumptions | Each spectrum was calibrated by setting the lysine cross peak (at 3.00– 1.67 ppm) and specifying a constant ‘number of contour levels’ (set to 28), as well as a constant ‘level multiplier’ (defined as the difference between values of consecutive contour, set to 1.05). |
| 4. Data Quality |  |
| a. Reported variables  (SNR, Linewidth (with reference peaks)) | *SNR and linewidth not described* |
| b. Data exclusion criteria | *No subjects excluded* |
| c. Quality measures of postprocessing Model fitting (e.g. CRLB, goodness of fit, SD of residual) | *No QA measures described* |
| d. Sample Spectrum | Figure 1 |

The example above used this following paper: Lin AP, Ramadan S, Stern RA, et al. Changes in the neurochemistry of athletes with repetitive brain trauma: preliminary results using localized correlated spectroscopy. Alzheimers Res Ther. 2015;7(1):13.

Items listed in italics are details that were not included in the paper that served as source for this example

**Appendix 2**. Example of the MRSinMRS checklist for a multi-sequence multi-nuclear MRS study (^1^H, ^31^P)

| **Site** (Name or Number) |  |  |  |
| --- | --- | --- | --- |
| 1. Hardware |  |  |  |
| a. Field strength [T] | 3T | 3T |  |
| b. Manufacturer | Siemens | Siemens |  |
| c. Model (software version if available) | Skyra (VD13) | Skyra (VD13) |  |
| d. RF coils: nuclei (transmit/ receive), number of channels, type, body part | 32 channel ^1^H head coil | *10 cm* ^31^P tuned transmit/receive  surface coil |  |
| e. Additional hardware | N/A | custom-built dynamic knee extension apparatus |  |
| 2. Acquisition |  |  |  |
| a. Pulse sequence | PRESS | 500-μs pulse and acquire |  |
| b. Volume of Interest (VOI) locations | Posterior cingulate gyrus | N/A |  |
| c. Nominal VOI size [cm^3^, mm^3^] | 30 x 30 x 30 mm^3^ | N/A |  |
| d. Repetition Time (TR), Echo Time (TE) [ms, s] | TR/TE = 2000/30 ms, | TR = 2000 ms |  |
| e. Total number of Excitations or acquisitions per spectrum  In time series for kinetic studies   1. Number of Averaged spectra (NA) per time-point 2. Averaging method (e.g. block-wise or moving average) 3. Total number of spectra (acquired / in time-series) | 64 averages | 300 total FIDS  SIFT used for averaging |  |
| f. Additional sequence parameters (spectral width in Hz, number of spectral points, frequency offsets)  If STEAM:, Mixing Time (TM)  If MRSI: 2D or 3D, FOV in all directions, matrix size, acceleration factors, sampling method | 1200 Hz, 1024 data points | 3000 Hz, 2048 data points |  |
| g. Water Suppression Method | CHESS | N/A |  |
| h. Shimming Method, reference peak, and thresholds for “acceptance of shim” chosen | Automated B0 field mapping followed by manual shimming of water to < 14 Hz | Automated B0 field mapping |  |
| i. Triggering or motion correction method  (respiratory, peripheral, cardiac triggering, incl. device used and delays) | None | None |  |
| 3. Data analysis methods and outputs |  |  |  |
| a. Analysis software | LCmodel vers 6.2 | jMRUI |  |
| b. Processing steps deviating from quoted reference or product | None | SIFT pre-processing prior to use of jMRUI |  |
| c. Output measure  (e.g. absolute concentration, institutional units, ratio)Processing steps deviating from quoted reference or product | Ratios to creatine | PCr amplitude |  |
| d. Quantification references and assumptions, fitting model assumptions | Default basis set | AMARES Gaussian lineshapes |  |
| 4. Data Quality |  |  |  |
| a. Reported variables  (SNR, Linewidth (with reference peaks)) | *SNR: 61.8 ± 6 (51-71)*  *FWHM: 0.043 + 0.006 (0.38-0.57) ppm as reported by LCmodel*  *None eliminated* | *SNR and FWHM not reported.* |  |
| b. Data exclusion criteria | *SNR< 40; CRLB>20%* | *SD>10%* |  |
| c. Quality measures of postprocessing Model fitting (e.g. CRLB, goodness of fit, SD of residual) | *CRLB of NAA: 2 ± 0(2)%* | *SD: 4.5 ± 1.7%* |  |
| d. Sample Spectrum | Figure 1 | Figure 2 |  |

The example above used this following paper: Zhou M, Liao H, Sreepada LP, Ladner JR, Balschi JA, Lin AP. Tai Chi Improves Brain Metabolism and Muscle Energetics in Older Adults. J Neuroimaging. 2018;28(4):359‐364. doi:10.1111/jon.12515

Items listed in italics are details that were not included in the paper that served as source for this example

**Appendix 3**: Example of the MRSinMRS checklist for an X-nuclear MRS study (dynamic ^31^P MRS, muscle)

| 1. Hardware |  |
| --- | --- |
| a. Field strength [T] | 7 T |
| b. Manufacturer | Siemens Healthineers, Erlangen, Germany |
| c. Model (software version if available) | Magnetom 7T (VB17) |
| d. RF coils: nuclei (transmit/receive), number of channels, type, body part | Custom-built three channel ^31^P (d = 15 cm, l = 10 cm), two channel ^1^H (d = 17 cm, l = 12.5 cm) transceiver coil, shaped to the human calf, (Ref: Goluch et al. Magn Reson Med 2015;73(6):1190–1195.) |
| e. Additional hardware | Custom-built pedal ergometer with pneumatic piston and MR-compatible sensors for pedal angle and force |
| 2. Acquisition |  |
| a. Pulse sequence | Semi-LASER |
| b. Volume of interest and VOI locations | Single voxel placed obliquely in gastrocnemius muscle, avoiding subcutaneous fat, fasciae and adjacent muscles |
| c. Nominal VOI size [cm^3^, mm^3^] | Anatomy-matched, 27 ± 6 cm^3^ *(ca. 2 ⨉ 3.5 ⨉ 4 cm^3^)* |
| d. Repetition Time (TR), Echo Time (TE) [ms, s] | TR = 6 s, TE = 29 ms |
| e. Total number of excitations or acquisitions per spectrum (NA)  In time series for kinetic studies   1. Number of averaged spectra) per time-point (NA) 2. Averaging method (e.g. block-wise or moving average)   Total number of spectra (acquired / in time-series) | *1 acquisition per spectrum (NA = 1). Except for pH quantification after 90 s post exercise, where NA = 4, with block-wise averaging. Total number of spectra in time series was 140, at NA = 1 (or series of 35 spectra at NA = 4).* |
| f. Additional sequence parameters (spectral width in Hz, number of spectral points, frequency offsets)   1. If STEAM:, Mixing Time (TM) 2. If MRSI: 2D or 3D, FOV in all directions, matrix size, acceleration factors, sampling method | 5 kHz, 2048 complex points after removing oversampling |
| g. Water suppression method | n.a. |
| h. Shimming method, reference peak, and thresholds for “acceptance of shim” chosen | *1^st^ and 2^nd^ order, vendor standard method (DESS sequence in “advanced shim” mode until convergence), line-width of PCr peak was evaluated post-hoc* |
| i. Triggering or motion correction method  (respiratory, peripheral, cardiac triggering, incl. device used and delays) | Subjects were instructed to push the pedal only during times without RF excitation or signal reception*, cued by gradient noise.* *Adherence to the protocol was inspected via data from the force sensors.* |
| 3. Data analysis methods and outputs |  |
| a. Analysis software | ^31^P MR spectroscopy data were processed  from raw data exported from the scanner using in-house developed Python scripts (http://www.python.org) for phasing and channel combination. Signals were phased to the highest peak magnitude of PCr in the frequency domain after 7 Hz Lorentzian apodization and 4 × zero-filling. The channel combination was then performed by weighted averaging of the raw data (that is, without apodization and zero-filling). Weights were calculated as proportional to signal, averaged over four resting  spectra (excluding the fully relaxed spectrum).  Spectra were then fitted in AMARES, as implemented in jMRUI, *version 5.0* |
| b. Processing steps deviating from quoted reference or product analysis software (vendor, version) | *Gaussian line shapes, soft constraints for frequencies* |
| c. Output measure  (e.g. absolute concentration, institutional units, ratio) Processing steps deviating from quoted reference or product | Concentrations in institutional units and pH values |
| d. Quantification references and assumptions, fitting model assumptions | *Quantification relative to total ^31^P signal, which was assumed to be constant.*  End-exercise PCr depletion relative to post-exercise asymptotic value of mono-exponential fit of recovery |
| 5. Data Quality |  |
| a. Reported variables  (SNR, Linewidth (with reference peaks)) | SNR was calculated using the partially saturated resting  spectra of each time series by dividing the PCr peak amplitude by the standard deviation of the signal in a region containing only noise, 15 ppm off-center across 1/16 of the total bandwidth. Linewidths were taken from the AMARES fit of the PCr peak. |
| b. Data exclusion criteria | *> 10 % changes of sum of total ^31^P signal*  *Linewidth of PCr peak > 15 Hz*  *Unphysiological pH values (> 7.1)*  *Splitting of Pi peak* |
| c. Quality measures of postprocessing Model fitting (e.g. CRLB, goodness of fit, SD of residual) | *SD of residual* |
| d. Sample Spectrum | Figure 2 |

The example above used this following paper: Niess F, Schmid AI, Bogner W, Wolzt M, Carlier PG, Trattnig S, Moser E, Meyerspeer M. Interleaved ^31^P MRS / ^1^H ASL for analysis of metabolic and functional heterogeneity along human lower leg muscles at 7T. Magn Reson Med 2020; 1909–1919. doi:10.1002/mrm.28088.

Items listed in italics are details that were not included in the paper that served as source for this example.

**Appendix 4**. Example of the multi-sequence MRSinMRS checklist for a combined single-voxel and magnetic resonance spectroscopic imaging study.

| 1. Hardware |  |  |  |
| --- | --- | --- | --- |
| a. Field strength [T] | 3 T | 3 T |  |
| b. Manufacturer | Siemens | Siemens |  |
| c. Model (software version if available) | Skyra (VD13B) | Skyra (VD13B) |  |
| d. RF coils: nuclei (transmit/ receive), number of channels, type, body part | 32 ch ^1^H head coil | 32 ch ^1^H head coil |  |
| e. Additional hardware | N/A | N/A |  |
| 2. Acquisition |  |  |  |
| a. Pulse sequence | PRESS | Semi-LASER CSI |  |
| b. Volume of Interest (VOI) locations | Patients: lesion  Controls: centrum semiovale | Patients: lesion |  |
| c. Nominal VOI size [cm^3^, mm^3^] | 20 x 20 x 20 mm^3^ | 80 x 80 x 15 mm^3^ |  |
| d. Repetition Time (TR), Echo Time (TE) [ms, s] | TR = 2000 ms, TE = 97ms | TR = 1700 ms, TE = 97 ms |  |
| e. Total number of Excitations or acquisitions per spectrum  In time series for kinetic studies   1. Number of Averaged spectra (NA) per time-point 2. Averaging method (e.g. block-wise or moving average) 3. Total number of spectra (acquired / in time-series) | 128 averages | 3 averages |  |
| f. Additional sequence parameters (bandwidth in Hz or dwell time in ms, number of spectral points, frequency offsets)  If STEAM:, Mixing Time (TM)  If MRSI: 2D or 3D, FOV in all directions, matrix size, acceleration factors, sampling method | 1200 Hz, 1024 points | 2D: 160 x 160 x 15 mm^3^ FOV; Matrix size: 16x16, no acceleration factor; weighted distribution sampling |  |
| g. Water Suppression Method | WET | WET |  |
| h. Shimming Method, reference peak, and thresholds for “acceptance of shim” chosen | automated 3D B0 field  mapping technique followed by manual adjustment < 14 Hz | automated 3D B0 field  mapping technique followed by manual adjustment < 25 Hz |  |
| i. Triggering or motion correction method  (respiratory, peripheral, cardiac triggering, incl. device used and delays) | N/A | N/A |  |
| 3. Data analysis methods and outputs |  |  |  |
| a. Analysis software | LCmodel 6.2 | LCmodel 6.2 |  |
| b. Processing steps deviating from quoted reference or product | Custom basis set | Custom basis set |  |
| c. Output measure  (e.g. absolute concentration, institutional units, ratio)Processing steps deviating from quoted reference or product | Ratios to creatine | Ratios to creatine |  |
| d. Quantification references and assumptions, fitting model assumptions | *The basis set included spectra of 2HG, NAA, GABA, glutamate, glycine, creatine, myo-inositol, glutamine, lactate, alanine, acetate, aspartate, ethanolamine, glutathione, phosphorylethanolamine, scyllo-inositol, taurine, N-acetylaspartylglutamate, glucose, and choline simulated using real pulses. Macromolecules were not modelled.* | *The basis set included spectra of 2HG, NAA, GABA, glutamate, glycine, creatine, myo-inositol, glutamine, lactate, alanine, acetate, aspartate, ethanolamine, glutathione, phosphorylethanolamine, scyllo-inositol, taurine, N-acetylaspartylglutamate, glucose, and choline simulated using real pulses. Macromolecules were not modelled.* |  |
| 4. Data Quality |  |  |  |
| a. Reported variables  (SNR, Linewidth (with reference peaks)) | *SNR and linewidths not reported* | *SNR and linewidths not reported* |  |
| b. Data exclusion criteria | SNR <5 or FWHM of Cr peak >0.143 ppm | 75^th^ percentile 2HG/Cr values of the selected voxels |  |
| c. Quality measures of postprocessing Model fitting (e.g. CRLB, goodness of fit, SD of residual) | 2HG CRLB < 30% | 2HG CRLB < 30% |  |
| d. Sample Spectrum | Figures 1-3 | Figures 1-3 |  |

The example above used this following paper: Zhou M, Zhou Y, Liao H, et al. Diagnostic accuracy of 2-hydroxyglutarate magnetic resonance spectroscopy in newly diagnosed brain mass and suspected recurrent gliomas. Neuro Oncol. 2018;20(9):1262-1271. doi:10.1093/neuonc/noy022

Items listed in italics are details that were not included in the paper that served as source for this example
